# Association between inflammatory markers and mortality in hemodialysis patients

**DOI:** 10.1590/2175-8239-JBN-2025-0312en

**Published:** 2026-05-11

**Authors:** Cigdem Cindoglu, Burcu Beyazgül, Derya Abuşka, Ufuk Acar

**Affiliations:** 1 Harran University, Faculty of Medicine, Department of Internal Medicine, Sanliurfa, Turkey.; 2 Harran University, Faculty of Medicine, Department of Public Health, Sanliurfa, Turkey.; 3 Istanbul Training and Research Hospital, Istanbul, Turkey.

**Keywords:** Renal Dialysis, Mortality, Biomarkers, Inflammation, Nutritional Status

## Abstract

**Introduction:**

This study aimed to evaluate the association between mortality and biomarkers reflecting inflammatory and nutritional status in hemodialysis (HD) patients, including the systemic immune-inflammation index (SII), neutrophil-to-lymphocyte ratio (NLR), platelet-to-lymphocyte ratio (PLR), C-reactive protein-to-albumin ratio (CAR), hemoglobin, albumin, lymphocyte and platelet (HALP) score, and prognostic nutritional index (PNI).

**Methods:**

This retrospective cohort study included 135 patients on routine HD. Baseline sociodemographic data, complete blood count, and biochemical parameters were recorded. Patients were followed for one year and classified as survivors or non-survivors. Predictors of mortality were analyzed using logistic regression (Enter method), with multicollinearity assessed by the variance inflation factor (VIF) and tolerance statistics.

**Results:**

The mean age of the patients included in the study was 55.2 ± 16.6 years, and 47.4% were male. In univariate analyses, age, albumin, calcium, CRP, white blood cell count, neutrophil count, hematocrit, hemoglobin, SII, NLR, CAR, and PNI were found to be significantly associated with survival (all p < 0.05). In the multivariate logistic regression analysis, only CAR (aOR = 1.374; 95% CI 1.011–1.869), PNI (aOR = 0.861; 95% CI 0.775–0.956), and hemoglobin (aOR = 0.725; 95% CI 0.532–0.988) were identified as independent predictors. The model’s accuracy rate was 82%, and the AUC value was calculated as 0.87.

**Conclusion:**

Our study demonstrated that easily accessible and low-cost parameters, particularly CAR and PNI, are clinically valuable in predicting survival. The identification of hemoglobin levels as an independent predictor further supports the prognostic significance of hematological parameters. This study may serve as a basis for future research on the prognostic use of these biomarkers, emphasizing the need for large-scale and prospective studies.

## Introduction

End-stage renal disease (ESRD) remains a major global health burden, particularly for individuals undergoing hemodialysis (HD). These patients carry an extremely high risk of mortality, with approximately 20% of cases resulting in death within the first year and nearly 60% within a five-year period of follow-up. Despite advances in dialysis technologies and care protocols, mortality rates in this population remain remarkably high. The leading causes of death among HD patients have been reported as cardiovascular diseases (53%), infections (18%), and discontinuation of dialysis therapy (16%)^1,2^.

Inflammation and malnutrition play a decisive role in the prognosis of HD patients, beyond their contributions as mortality risk factors^3^. Inflammatory and nutritional biomarkers obtained from routine blood tests are increasingly recognized as important tools for predicting prognosis in various diseases. However, it remains unclear which biomarkers carry the strongest prognostic value in HD patients^4^. The systemic immune-inflammation index (SII) was first introduced by Hu et al.^5^ in 2014 and was developed based on platelet, neutrophil, and lymphocyte counts. Systemic inflammation in HD patients is recognized as a key pathophysiological mechanism closely associated with increased mortality. Moreover, SII has been shown to be an independent risk factor for protein-energy wasting in maintenance HD patients^6,7^. The neutrophil-to-lymphocyte ratio (NLR), which can be easily calculated from the inexpensive and widely used complete blood count, has been strongly associated with mortality in HD patients due to its link with systemic inflammation and endothelial damage. In contrast, the relationship between the platelet-to-lymphocyte ratio (PLR) and mortality has been reported to be weaker^8^.

The C-reactive protein-to-albumin ratio (CAR) has been used as a practical biomarker reflecting both inflammatory status and nutritional conditions^9^. Previous studies have associated higher CRP levels or lower serum albumin concentrations with an increased incidence of contrast-induced nephropathy and mortality^10^. A recent study demonstrated that CAR is an effective, simple, and low-cost biomarker for predicting early mortality in HD patients^11^. The HALP score (hemoglobin × albumin × lymphocyte / platelet) was developed by Chen et al.^12^ to predict prognosis in gastric carcinoma. In HD patients, a study reported a negative association between baseline HALP values and both all-cause and cardiovascular mortality, suggesting that low pre-dialysis HALP levels may serve as a reliable indicator of poor prognosis^13^. The prognostic nutritional index (PNI), calculated from serum albumin levels and total lymphocyte count, has been proposed as a simple and practical screening tool for predicting prognosis in cancer patients^14^. Studies in HD patients have reported that higher PNI values are associated with reduced mortality. Notably, in patients under 65 years of age, it has been shown to be a stronger predictor of one-year mortality than either serum albumin or total lymphocyte count alone^15^.

Findings from the literature suggest that biomarkers reflecting inflammatory and nutritional status may be associated with mortality in HD patients; however, the evidence evaluating these parameters simultaneously remains limited. Therefore, the aim of this study was to examine the associations between selected, easily accessible, low-cost inflammatory and nutritional indices (SII, NLR, PLR, CAR, HALP, and PNI) and mortality in HD patients, as well as to identify which of these parameters remain associated with survival after multivariable adjustment.

## Methods

Data for this retrospective, single-center cohort study were obtained from routine HD patients followed at the Dialysis Clinic of Harran University Faculty of Medicine Hospital between January 2022 and January 2023. All HD patients treated at the center during the study period were initially assessed. A total of five patients were excluded due to concomitant malignancy, active infection at baseline, or pregnancy. The remaining 135 HD patients were consecutively enrolled in the study without additional sampling.

Sociodemographic characteristics, including age, sex, and HD vintage, as well as baseline hematological and biochemical parameters recorded at the initiation of dialysis, were extracted from medical records. Blood samples were obtained as part of routine clinical care prior to the HD session, following an overnight fasting period. All laboratory analyses were performed in the hospital’s central laboratory using standardized and routinely calibrated automated analyzers. For each patient, a single baseline laboratory measurement obtained at the start of the dialysis period was used for analysis. Composite indices, including CAR, SII, and PNI, were calculated based on these baseline values. At the end of the one-year follow-up period, patients were classified as deceased or survivors, and associations between selected inflammatory and nutritional indices and mortality were evaluated. The dependent variable of the study was defined as mortality status (deceased vs. survivor). Sociodemographic characteristics, as well as hematological and biochemical parameters considered potential determinants of this outcome, were included as independent variables.

Data analysis was performed using IBM SPSS Statistics v.22.0 (IBM Corp.; Armonk, NY, USA). Descriptive statistics, including mean, standard deviation, minimum, maximum, and percentages, were used to evaluate the study data. The normality of the data distribution was assessed using skewness, kurtosis, and the Kolmogorov–Smirnov test. Since the data were normally distributed, the independent-samples t-test was used for univariate analyses, while categorical variables were analyzed using the chi-square test. Multivariate analyses were performed using logistic regression with the Enter method. Multicollinearity was evaluated using the variance inflation factor (VIF) and tolerance statistics. Ethical approval was obtained from the Clinical Research Ethics Committee (07.08.2023; HRÜ/23.14.19), and institutional permission granted by the Dean’s Office of the Harran University Faculty of Medicine.

## Results

The mean age of the individuals was 55.23 ± 16.56 years. Among the patients, 47.4% (64 individuals) were male, and 54.81% (74 individuals) had at least one comorbidity. The most common comorbidities were diabetes mellitus (DM) in 34.7% (46 patients) and hypertension (HT) in 31.11% (42 patients). The distribution of the study group’s sociodemographic, clinical, and laboratory characteristics is presented in detail in Table 1. When survival status was analyzed according to sex and the presence of comorbidities, no significant differences were observed (Table 2; p = 0.181 and p = 0.346, respectively).

**Table 1:** Distribution of sociodemographic, clinical, and laboratory parameters of the study group

Parameters	Values
Age (years), mean ± SD (min–max)	55.23 ± 16.56 (20–84)
Sex	
Male (N/%)	64 (47.4)
Female (N/%)	71 (52.6)
Presence of comorbidities	
DM (N/%)	46 (34.07)
Hypertension (HT) (N/%)	42 (31.11)
Cardiac disease (N/%)	15 (11.11)
Others (N/%)	7 (5.19)
Number of comorbidities	
None	61 (45.19)
1	46 (34.07)
2	20 (14.81)
≥3	8 (5.93)
Kt/V, mean ± SD (min–max)	1.71 ± 0.33 (0.95–3.06)
Hemoglobin (g/dL)	10.62 ± 1.70 (6.37–15.40)
Hematocrit (%)	33.61 ± 5.39 (20.70–50.10)
WBC (×10^3^/µL)	8.67 ± 4.90 (2.53–32.50)
Neutrophils (×10^3^/µL)	5.86 ± 4.39 (1.73–30.06)
Lymphocytes (×10^3^/µL)	1.61 ± 0.86 (0.20-7.90)
Platelets (×10^3^/µL)	211.38 ± 82.94 (8.58–526.00)
Glucose (mg/dL)	140.96 ± 102.43 (60.00–951.00)
Urea (mg/dL)	112.12 ± 42.32 (15.00–351.00)
Creatinine (mg/dL)	7.38 ± 5.41 (0.70–18,50)
Uric acid (mg/dL)	6.24 ± 1.49 (1.90–10.60)
ALT (U/L)	16.74 ± 21.86 (1.00–229.00)
Albumin (g/dL)	3.62 ± 0.75 (1.80–5.30)
Na (mmol/L)	134.66 ± 11.20 (14.00–146.00)
K (mmol/L)	5.72 ± 5.31 (3.00–7.70)
Ca (mg/dL)	8.40 ± 0.89 (5.10–10.50)
CRP (mg/L)	3.75 ± 5.96 (0.05–34.00)
TSH (µIU/mL)	15.05 ± 46.39 (0.12–491.00)
PTH (pg/mL)	686.00 ± 491.63 (32.30–2000.00)
Ferritin (ng/mL)	562.11 ± 568.08 (8.30–4511.00)
Iron (µg/dL)	48.42 ± 21.75 (9.00–142.00)
SII	1137.89 ± 2983.13 (0.00–33857.05)
NLR	5.02 ± 8.07 (0.25–79.11)
PLR	157.77 ± 115.57 (4.09–1126.32)
CAR	1.20 ± 2.06 (0.01–11.90)
HALP	0.41 ± 1.10 (0.00–12.77)
PNI	36.16 ± 7.60 (28.00–90.00)

Abbreviations – DM: diabetes mellitus; HT: hypertension; WBC: white blood cell count; ALT: alanine aminotransferase; Na: sodium; K: potassium; Ca: calcium; CRP: C-reactive protein; TSH: thyroid-stimulating hormone; PTH: parathyroid hormone; SII: systemic immune-inflammation index; NLR: neutrophil-to-lymphocyte ratio; PLR: platelet-to-lymphocyte ratio; CAR: C-reactive protein-to-albumin ratio; HALP: hemoglobin × albumin × lymphocytes / platelets; PNI: prognostic nutritional index.

**Table 2:** Survival outcomes according to patient age and sex

Parameters		Survivors		Mortality	
		N	%	N	%
Sex	Female	53	74.6	18	25.4
	Male	40	62.5	20	37.5
				X^2^: 1.785	p = 0.181
Comorbidities	Present	54	73.0	20	27.0
	Absent	39	63.9	22	36.1
				X^2^: 0.888	p = 0.346

Univariate analyses of laboratory parameters potentially affecting patient survival are presented in Table 3. Age, albumin, calcium (Ca), C-reactive protein (CRP), white blood cell count (WBC), neutrophils, hematocrit, hemoglobin, parathyroid hormone (PTH), SII, NLR, CAR, and PNI were found to be statistically significant (p = 0.019; p = 0.008; p = 0.031; p <0.001; p <0.001; p = 0.001; p = 0.022; p = 0.001; p = 0.012; p = 0.016; p = 0.016; p <0.001; and p = 0.010, respectively).

**Table 3:** Distribution of survival according to laboratory parameters of the patients

Parameter	Survival status	N	Mean ± SD	t	df	p
Age (years)	Survival	93	53.16 ± 17.34	–2.389	**98.4**	**0.019**
	Mortality	42	59.81 ± 13.76			
Kt/V	Survival	93	1.72 ± 0.36	0.580	133.0	0.563
	Mortality	42	1.69 ± 0.27			
Glucose (mg/dL)	Survival	93	135.53 ± 110.56	–0.917	133.0	0.361
	Mortality	42	153.00 ± 81.54			
Urea (mg/dL)	Survival	93	113.28 ± 35.97	0.470	133.0	0.639
	Mortality	42	109.57 ± 54.23			
Creatinine (mg/dL)	Survival	93	7.41 ± 2.86	0.066	45.0	0.948
	Mortality	42	7.32 ± 8.79			
Uric acid (mg/dL)	Survival	93	6.23 ± 1.51	-0.044	133.0	0.965
	Mortality	42	6.25 ± 1.44			
ALT (U/L)	Survival	93	14.80 ± 12.13	–1.123	44.26	0.268
	Mortality	42	21.12 ± 35.12			
Albumin (g/dL)	Survival	93	3.76 ± 0.48	2.745	**48.5**	**0.008**
	Mortality	42	3.29 ± 1.08			
Na (mmol/L)	Survival	93	133.98 ± 13.15	–1.051	133.0	0.295
	Mortality	42	136.17 ± 4.30			
K (mmol/L)	Survival	93	5.35 ± 0.89	–0.813	41.3	0.421
	Mortality	42	6.54 ± 9.44			
Ca (mg/dL)	Survival	93	8.51 ± 0.90	2.185	**133.0**	**0.031**
	Mortality	42	8.15 ± 0.84			
Iron (µg/dL)	Survival	93	47.94 ± 22.46	–0.382	133.0	0.703
	Mortality	42	49.49 ± 20.29			
CRP (mg/L)	Survival	93	1.98 ± 3.74	–4.455	**49.6**	**<0.001**
	Mortality	42	7.66 ± 7.87			
WBC (×10^3^/µL)	Survival	93	7.32 ± 2.57	–3.848	**45.9**	**<0.001**
	Mortality	42	11.65 ± 7.09			
Lymphocyte count (×10^3^/µL)	Survival	93	1.60 ± 0.67	–0.240	133.0	0.811
	Mortality	42	1.64 ± 1.18			
Neutrophil count (×10^3^/µL)	Survival	93	4.71 ± 2.05	–3.537	**44.6**	**0.001**
	Mortality	42	8.40 ± 6.62			
Hematocrit (%)	Survival	93	34.32 ± 5.30	2.320	**133.0**	**0.022**
	Mortality	42	32.03 ± 5.30			
Hemoglobin (g/dL)	Survival	93	10.94 ± 1.67	3.342	**133.0**	**0.001**
	Mortality	42	9.92 ± 1.57			
Platelet count (×10^3^/µL)	Survival	93	214.00 ± 73.93	0.544	133.0	0.587
	Mortality	42	205.58 ± 100.82			
TSH (µIU/mL)	Survival	93	17.22 ± 79.81	0.542	51.0	0.590
	Mortality	42	0.73 ± 0.36			
PTH (pg/mL)	Survival	93	744.49 ± 546.75	2.545	**126.7**	**0.012**
	Mortality	42	556.48 ± 306.91			
Ferritin (ng/mL)	Survival	93	521.02 ± 479.11	–1.253	133.0	0.212
	Mortality	42	653.12 ± 726.19			
SII	Survival	93	725.32 ± 585.21	–2.435	**133.0**	**0.016**
	Mortality	42	2051.44 ± 5203.40			
NLR	Survival	93	3.38 ± 2.63	–2.518	**42.47**	**0.016**
	Mortality	42	8.60 ± 13.30			
PLR	Survival	93	150.29 ± 73.29	–0.846	47.60	0.400
	Mortality	42	174.17 ± 176.08			
CAR	Survival	93	0.58 ± 1.18	–4.511	**38.45**	**<0.001**
	Mortality	42	2.84 ± 2.87			
HALP	Survival	93	0.45 ± 1.30	0.655	130.0	0.514
	Mortality	42	0.32 ± 0.27			
PNI	Survival	93	37.65 ± 4.81	2.708	**44.05**	**0.010**

Abbreviations – ALT: alanine aminotransferase; Na: sodium; K: potassium; Ca: calcium; CRP: C-reactive protein; WBC: white blood cell count, TSH: thyroid-stimulating hormone; PTH: parathyroid hormone; SII: systemic immune-inflammation index; NLR: neutrophil-to-lymphocyte ratio; PLR: platelet-to-lymphocyte ratio; CAR: C-reactive protein-to-albumin ratio; HALP: hemoglobin × albumin × lymphocytes / platelets; PNI: prognostic nutritional index.

In line with the study objective, the association between easily accessible indices reflecting inflammatory and nutritional status and mortality in HD patients was evaluated using multivariable logistic regression analysis. Variables included in the multivariable model were selected based on clinical relevance and statistical significance in univariate analyses. The model was adjusted for sex and the presence of comorbidities.

Prior to model construction, multicollinearity among candidate variables was assessed using correlation matrices, the VIF, and tolerance statistics. To reduce redundancy and improve model stability, composite indices (such as PNI and CAR) and their individual components (e.g., albumin, CRP, WBC) were not entered simultaneously into the same model, and collinear variables were excluded accordingly.

Given the limited sample size, the findings of the multivariable model should be interpreted as associative rather than predictive, and no formal assessment of diagnostic or predictive performance was conducted.

In the final model, CAR was positively associated with mortality (adjusted odds ratio [aOR] = 1.374; 95% confidence interval [CI] 1.011–1.869). PNI and hemoglobin levels were negatively associated with mortality: PNI, aOR = 0.861 (95% CI 0.775–0.956); hemoglobin, aOR = 0.725 (95% CI 0.532–0.988). No significant associations were observed between age (aOR = 1.011; 95% CI 0.980–1.043) or WBC (aOR = 1.110; 95% CI 0.943–1.307) and mortality. The overall model fit was statistically significant (χ^2^(5) = 51.0, p < 0.001), with a Nagelkerke R^2^ of 0.456 (Table 4). Using the established cutoff point, the positive class was defined as “death.” The model correctly classified 91.4% of survivors (85/93) and 59.5% of deaths (25/42), with an overall accuracy of 82% (0.82). Sensitivity was 0.60, specificity was 0.91, and the area under the curve (AUC) was 0.87 (Table 5).

**Table 4:** Binary logistic regression analysis of parameters predicting mortality

Predictor	β	p-value	aOR	95% CI	
				Lower	Upper
Intercept	5.834	0.023	341.625	2.221	52547.153
Age (years)	0.011	0.487	1.011	0.980	1.043
CAR	**0.318**	**0.043**	**1.374**	**1.011**	**1.869**
PNI	**–0.151**	**0.005**	**0.861**	**0.775**	**0.956**
Hemoglobin (g/dL)	**–0.322**	**0.042**	**0.725**	**0.532**	**0.988**
White blood cell count (×10^9^/L)	0.104	0.210	1.110	0.943	1.307
**Model fit measures:** Deviance: 109 **Pseudo R** ^2^ Nagelkerke’s: 0.456 **Overall model test** [χ^2^(df), p]: 51.0 (5), <0.001					

Abbreviations – CAR: C-reactive protein-to-albumin ratio; PNI: prognostic nutritional index.

Notes – Dependent variable: mortality (non-survivors: 42; survivors [reference]: 93). Model estimated with N = 135. aOR = adjusted odds ratio; aORs were adjusted for sex and presence of comorbidities (control variables).

**Table 5:** Predictive performance of the developed model (classification table)

Observed	Predicted		Accuracy (%)
	Survivors	Non-survivors	
Survivors	85	8	91.4
Non-survivors	17	25	59.5
Overall accuracy	0.82		
Specificity	0.91		
Sensitivity	0.60		
AUC	0.87		

## Discussion

In this study, the relationship between clinical and laboratory parameters and survival was examined in 135 patients undergoing HD treatment. Univariate analyses revealed significant associations between survival and age, albumin, calcium, CRP, WBC, neutrophils, hematocrit, hemoglobin, PTH, SII, NLR, CAR, and PNI levels. In the multivariate logistic regression analysis, CAR, PNI, and hemoglobin emerged as independent predictors of survival. While low hemoglobin levels were associated with increased mortality, higher CAR and lower PNI levels were linked to higher mortality risk. The established model demonstrated clinically meaningful predictive performance, with high specificity (91%) and overall accuracy (82%).

SII has recently emerged as a notable biomarker for predicting mortality in HD patients. Studies have reported that high SII levels are independently associated with both all-cause mortality and mortality due to cardiovascular disease and infections^16,17^.

Additionally, several studies have compared multiple inflammatory-nutritional indices in HD populations, reporting that SII may demonstrate relatively stronger prognostic performance for mortality when evaluated alongside indices such as NLR, PLR, and CAR. However, evidence from smaller retrospective cohorts assessing these indices concurrently suggests that their predictive value is not consistent across populations and should be interpreted as exploratory and cohort-specific rather than universally applicable^18,19^.

In our study, SII was associated with survival in univariate analyses but did not emerge as an independent predictor in multivariate analyses. Although the independent prognostic value of SII has been frequently emphasized in the literature, this discrepancy may be attributed to differences in patient profiles, sample characteristics, or variables included in the model.

Inflammation plays a significant role in malnutrition among HD patients, and therefore, CAR serves as a more dynamic marker by reflecting both inflammatory processes and protein-energy wasting. A study by Yang et al.^20^ demonstrated that CAR outperforms other biomarkers, including albumin, in predicting systemic inflammation and related complications. Moreover, the literature highlights CAR as a potentially relevant prognostic indicator for mortality in HD patients. In a retrospective cohort of incident HD patients, higher CAR levels were reported to be independently associated with increased one-year mortality, supporting the role of the imbalance between inflammation and nutrition in survival outcomes^21^. In our study, CAR also emerged as an independent predictor in multivariate analyses and was among the strongest determinants of survival. These findings are consistent with previous reports and suggest that CAR represents a valuable prognostic marker for clinical use in the HD population.

NLR and PLR are easily accessible biomarkers of the inflammatory response, and their associations with mortality in HD patients have been examined in various studies. These investigations have reported that both high NLR and high PLR values are significantly associated with poorer survival^22^. In our study, NLR was significantly associated with survival in univariate analyses but did not retain independent prognostic significance after multivariate adjustment, while PLR was not associated with survival in either univariate or multivariate analyses. Although previous studies have reported associations between elevated NLR and PLR levels and poorer survival outcomes in HD populations, our findings suggest that the prognostic contribution of these indices may be context dependent. This effect may be attenuated when stronger clinical and laboratory variables are considered^23^.

In a prospective study evaluating approximately 763 incident HD patients, those with low HALP scores were found to have a significantly higher risk of both cardiovascular events and all-cause mortality compared to patients with high scores^24^. In addition to other inflammatory-nutritional indices, the HALP score has been explored in maintenance HD populations, with retrospective studies suggesting a potential association between higher values and lower all-cause mortality. However, this association was not observed in our study, which may be related to differences in patient profiles, comorbidity burden, or clinical characteristics, highlighting the need for cohort-specific validation of HALP as a prognostic marker^25^. Similarly, another recent study reported that low PNI levels were associated with increased five-year mortality risk in HD patients and suggested that this index could serve as an independent predictor of long-term survival^26^. In our work, PNI levels were significantly associated with survival, with higher PNI values linked to a reduced risk of mortality. These findings are consistent with previous reports and support the notion that PNI represents an important biomarker to consider in the prognostic assessment of HD patients.

In this population, hemoglobin categories of <9.0, 9.0–9.9, and ≥13.0 g/dL have been associated with significantly increased mortality risk compared to the reference range of 10.0–10.9 g/dL. Additionally, the 12.0–12.9 g/dL category may also confer elevated risk depending on the patient’s clinical history (e.g., certain comorbid conditions). These findings support the notion that an approximate hemoglobin range of 10–12 g/dL is optimal in HD patients^27,28^. In our cohort, hemoglobin levels were significantly higher among survivors (10.94 ± 1.67 g/dL) and lower in those who died (9.92 ± 1.57 g/dL; t = 3.342, df = 133, p = 0.001). The mean difference of approximately 1.0 g/dL indicates that the deceased group was closer to the <10 g/dL threshold, whereas survivors fell within the 10.0–10.9 g/dL band. When our data are considered alongside published findings, the evidence linking low hemoglobin levels (<10 g/dL) with increased mortality is reinforced. These results suggest that, in clinical practice, targeting a hemoglobin range of approximately 10–12 g/dL is rational, avoiding overcorrection (≥13 g/dL) and accounting for comorbidities. Although our sample did not include sufficient patients to evaluate risks at the upper end, the available data clearly support the risk associated with lower hemoglobin levels.

The limitations of our study include its retrospective design, single-center setting, and relatively small sample size, which restrict the generalizability of the findings and introduce the potential for selection bias. Additionally, some parameters (e.g., PTH, ALP) were excluded from analysis due to either lack of routine measurement or variability related to treatment. Furthermore, the retrospective, single-center cohort design precluded assessment of temporal changes in biomarkers and did not allow for the determination of causal relationships. Finally, only baseline values were considered, and the impact of dynamic changes in biomarkers during long-term follow-up on prognosis could not be evaluated.

## Conclusion

In conclusion, the present study found that CAR, PNI, and hemoglobin levels were associated with survival in multivariable logistic regression analyses. These findings suggest that biomarkers reflecting inflammation, nutritional status, and hematological parameters may be related to prognosis in HD patients. The potential utility of routinely available and easily obtainable parameters such as CAR and PNI in prognostic assessment is noteworthy. However, considering the study design and sample size, further large-scale prospective studies are required to clarify the role of these biomarkers in clinical decision-making.

## Data Availability

The full dataset supporting the findings of this study is available upon request from the corresponding author, Çiğdem Cindoğlu. The dataset is not publicly available due to the presence of sensitive clinical information that could compromise participant privacy.
