# Supplementary material for: Association between inflammatory markers and mortality in hemodialysis patients
Source: J Bras Nefrol. 2026 May 11;48(3):e20250312. doi: 10.1590/2175-8239-JBN-2025-0312en (PMC13166101; doi:10.1590/2175-8239-JBN-2025-0312en)
Supplement: Translation

# Translation: Associação entre marcadores inflamatórios e mortalidade em pacientes em hemodiálise

**DOI:** 10.1590/2175-8239-JBN-2025-0312pt

**Authors:** Cigdem Cindoglu, Burcu Beyazgül, Derya Abuşka, Ufuk Acar

**Affiliations:** 1 Harran University, Faculty of Medicine, Department of Internal Medicine, Sanliurfa, Turkey.; 2 Harran University, Faculty of Medicine, Department of Public Health, Sanliurfa, Turkey.; 3 Istanbul Training and Research Hospital, Istanbul, Turkey.

**Keywords:** Diálise Renal, Mortalidade, Biomarcadores, Inflamação, Estado Nutricional

## Abstract

**Objetivo:**

Avaliar a associação entre mortalidade e biomarcadores que refletem o estado inflamatório e nutricional em pacientes em hemodiálise (HD), incluindo o índice de inflamação imune sistêmica (SII), a razão neutrófilos/linfócitos (NLR), a razão plaqueta/linfócito (PLR), a razão proteína C-reativa/albumina (CAR), hemoglobina, albumina, linfócitos e plaquetas (HALP) e o índice prognóstico nutricional (PNI).

**Métodos:**

Estudo de coorte retrospectivo que incluiu 135 pacientes em HD de rotina. Foram registrados dados sociodemográficos basais, hemograma e parâmetros bioquímicos. Os pacientes foram acompanhados por um ano e classificados como sobreviventes ou não sobreviventes. Os preditores de mortalidade foram analisados por regressão logística (método *Enter*), com a multicolinearidade avaliada pelo fator de inflação da variância (VIF) e por estatísticas de tolerância.

**Resultados:**

A idade média dos pacientes incluídos no estudo foi de 55,2 ± 16,6 anos, e 47,4% eram do sexo masculino. Nas análises univariadas, idade, albumina, cálcio, PCR, contagem de leucócitos, contagem de neutrófilos, hematócrito, hemoglobina, SII, NLR, CAR e PNI foram significativamente associados à sobrevida (todos p < 0,05). Na análise de regressão logística multivariada, apenas CAR (aOR = 1,374; IC 95% 1,011–1,869), PNI (aOR = 0,861; IC 95% 0,775–0,956) e hemoglobina (aOR = 0,725; IC 95% 0,532–0,988) foram identificados como preditores independentes. A taxa de acurácia do modelo foi de 82%, e o valor da AUC foi calculado como 0,87.

**Conclusão:**

Demonstrou-se que parâmetros de fácil acesso e baixo custo, particularmente CAR e PNI, são clinicamente valiosos na predição da sobrevida. A identificação do nível de hemoglobina como preditor independente reforça ainda mais a significância prognóstica dos parâmetros hematológicos. Este estudo pode servir como base para pesquisas futuras sobre o uso prognóstico desses biomarcadores, enfatizando a necessidade de estudos prospectivos em maior escala.

## Introdução

A doença renal em estágio terminal (DRET) permanece como um importante problema de saúde global, particularmente em indivíduos submetidos à hemodiálise (HD). Esses pacientes apresentam um risco extremamente elevado de mortalidade, com aproximadamente 20% dos casos evoluindo a óbito no primeiro ano e quase 60% em cinco anos de acompanhamento. Apesar dos avanços nas tecnologias de diálise e nos protocolos de cuidado, as taxas de mortalidade nessa população permanecem notavelmente altas. As principais causas de morte entre pacientes em HD têm sido relatadas como doenças cardiovasculares (53%), infecções (18%) e interrupção da terapia dialítica (16%)^1,2^.

A inflamação e a desnutrição desempenham um papel decisivo no prognóstico de pacientes em HD, além de suas contribuições como fatores de risco para mortalidade^3^. Biomarcadores inflamatórios e relacionados ao estado nutricional, obtidos a partir de exames laboratoriais de rotina, têm sido cada vez mais reconhecidos como ferramentas importantes para a predição do prognóstico em diversas doenças. No entanto, ainda não está claro quais biomarcadores apresentam o maior valor prognóstico em pacientes em HD^4^. O índice de inflamação imune sistêmica (SII) foi introduzido pela primeira vez por Hu et al.^5^, em 2014, e desenvolvido com base nas contagens de plaquetas, neutrófilos e linfócitos. A inflamação sistêmica em pacientes em HD é reconhecida como um mecanismo fisiopatológico chave, intimamente associado ao aumento da mortalidade. Além disso, o SII demonstrou ser um fator de risco independente para o *wasting* proteico-energético (*protein-energy wasting*) em pacientes em HD de manutenção^6,7^. A razão neutrófilos/linfócitos (NLR), que pode ser facilmente calculada a partir do hemograma completo, exame amplamente utilizado e de baixo custo, tem sido fortemente associada à mortalidade em pacientes em HD devido à sua relação com a inflamação sistêmica e o dano endotelial. Em contraste, a relação entre a razão plaqueta/linfócito (PLR) e a mortalidade tem sido relatada como mais fraca^8^.

A razão proteína C-reativa/albumina (CAR) tem sido utilizada como um biomarcador prático que reflete tanto o estado inflamatório quanto a condição nutricional^9^. Estudos prévios associaram níveis mais elevados de PCR ou concentrações séricas mais baixas de albumina a uma maior incidência de nefropatia induzida por contraste e à mortalidade^10^. Um estudo recente demonstrou que a CAR é um biomarcador eficaz, simples e de baixo custo para a predição da mortalidade precoce em pacientes em HD^11^. O escore HALP (hemoglobina × albumina × linfócitos / plaquetas) foi desenvolvido por Chen et al.^12^ para avaliar o prognóstico em carcinoma gástrico. Em pacientes em HD, um estudo relatou associação negativa entre os valores basais de HALP e tanto a mortalidade por todas as causas quanto a mortalidade cardiovascular, sugerindo que baixos níveis de HALP pré-diálise podem servir como um indicador confiável de mau prognóstico^13^. O índice prognóstico nutricional (PNI), calculado a partir dos níveis séricos de albumina e da contagem total de linfócitos, tem sido proposto como uma ferramenta de triagem simples e prática para a predição do prognóstico em pacientes com câncer^14^. Estudos em pacientes em HD relataram que valores mais elevados de PNI estão associados à menor mortalidade e que, particularmente em pacientes com menos de 65 anos, o PNI atua como um preditor mais forte de mortalidade em um ano do que a albumina sérica ou a contagem total de linfócitos isoladamente^15^.

Evidências da literatura sugerem que biomarcadores que refletem o estado inflamatório e nutricional podem estar associados à mortalidade em pacientes em HD; entretanto, as evidências que avaliam esses parâmetros de forma simultânea ainda são limitadas. Dessa forma, o objetivo deste estudo foi examinar as associações entre índices inflamatórios e nutricionais selecionados, de fácil acesso e de baixo custo (SII, NLR, PLR, CAR, HALP e PNI), e a mortalidade em pacientes em HD, bem como identificar quais desses parâmetros permanecem associados à sobrevida após ajuste multivariável.

## Métodos

Os dados deste estudo de coorte retrospectivo, de centro único, foram obtidos a partir de pacientes em HD de rotina, acompanhados na Clínica de Diálise do Hospital da Faculdade de Medicina da Universidade de Harran, entre janeiro de 2022 e janeiro de 2023. Todos os pacientes em HD tratados no centro durante o período do estudo foram inicialmente avaliados. Cinco indivíduos foram excluídos devido à presença de malignidade concomitante, infecção ativa no início do acompanhamento ou gravidez. Os 135 pacientes em HD remanescentes foram incluídos consecutivamente no estudo, sem amostragem adicional.

As características sociodemográficas, incluindo idade, sexo e tempo em HD, bem como os parâmetros hematológicos e bioquímicos basais registrados no início da diálise, foram extraídos dos prontuários médicos. As amostras de sangue foram coletadas como parte do cuidado clínico de rotina, antes da sessão de HD, após um período de jejum noturno. Todas as análises laboratoriais foram realizadas no laboratório central do hospital, utilizando analisadores automatizados padronizados e calibrados rotineiramente. Para cada paciente, utilizou-se uma única medida laboratorial basal para análise, obtida no início do período dialítico. Índices compostos, incluindo CAR, SII e PNI, foram calculados com base nesses valores basais. Ao final do período de seguimento de um ano, os pacientes foram classificados como falecidos ou sobreviventes, e as associações entre os índices inflamatórios e nutricionais selecionados e a mortalidade foram avaliadas. A variável dependente do estudo foi definida como o estado de mortalidade (sobrevivente vs. óbito). As características sociodemográficas, bem como os parâmetros hematológicos e bioquímicos considerados potenciais influenciadores desse desfecho, foram definidos como variáveis independentes.

A análise dos dados foi realizada utilizando-se o software IBM SPSS *Statistics* versão 22.0 (IBM Corp.; Armonk, NY, EUA). Estatísticas descritivas, incluindo média, desvio padrão, mínimo, máximo e percentual, foram utilizadas para a avaliação dos dados do estudo. A normalidade da distribuição dos dados foi avaliada por meio da assimetria, da curtose e do teste de *Kolmogorov–Smirnov*. Como os dados apresentaram distribuição normal, o teste t de *Student* para amostras independentes foi utilizado nas análises univariadas, enquanto as variáveis categóricas foram analisadas por meio do teste do qui-quadrado. As análises multivariadas foram realizadas por regressão logística, por meio do método *Enter*. A multicolinearidade foi avaliada por meio do fator de inflação da variância (VIF) e das estatísticas de tolerância. A aprovação ética foi obtida junto ao Comitê de Ética em Pesquisa Clínica (07.08.2023; HRÜ/23.14.19), e a autorização institucional foi concedida pelo Decanato da Faculdade de Medicina da Universidade de Harran.

## Resultados

A idade média dos indivíduos foi de 55,23 ± 16,56 anos. Entre os pacientes, 47,4% (64 indivíduos) eram do sexo masculino, e 54,81% (74 indivíduos) apresentavam pelo menos uma comorbidade. As comorbidades mais frequentes foram diabetes mellitus (DM) em 34,7% (46 pacientes) e hipertensão arterial (HA) em 31,11% (42 pacientes). A distribuição das características sociodemográficas, clínicas e laboratoriais do grupo estudado foi apresentada em detalhes na Tabela 1. Não foram observadas diferenças significativas no estado de sobrevida quando analisado segundo o sexo e a presença de comorbidades (Tabela 2; p = 0,181 e p = 0,346, respectivamente).

**Tabela 1:** Distribuição dos parâmetros sociodemográficos, clínicos e laboratoriais do grupo de estudo

Parâmetros	Valores
Idade (anos), média ± DP (mín.–máx.)	55,23 ± 16,56 (20–84)
Sexo	
Masculino (N/%)	64 (47,4)
Feminino (N/%)	71 (52,6)
Presença de comorbidades	
DM (N/%)	46 (34,07)
Hipertensão arterial (HA) (N/%)	42 (31,11)
Doença cardíaca (N/%)	15 (11,11)
Outras (N/%)	7 (5,19)
Número de comorbidades	
Nenhuma	61 (45,19)
1	46 (34,07)
2	20 (14,81)
≥3	8 (5,93)
Kt/V, média ± DP (mín.–máx.)	1,71 ± 0,33 (0,95–3,06)
Hemoglobina (g/dL)	10,62 ± 1,70 (6,37–15,40)
Hematócrito (%)	33,61 ± 5,39 (20,70–50,10)
Leucócitos (WBC) (×10^3^/µL)	8,67 ± 4,90 (2,53–32,50)
Neutrófilos (×10^3^/µL)	5,86 ± 4,39 (1,73–30,06)
Linfócitos (×10^3^/µL)	1,61 ± 0,86 (0,20–7,90)
Plaquetas (×10^3^/µL)	211,38 ± 82,94 (8,58–526,00)
Glicose (mg/dL)	140,96 ± 102,43 (60,00–951,00)
Ureia (mg/dL)	112,12 ± 42,32 (15,00–351,00)
Creatinina (mg/dL)	7,38 ± 5,41 (0,70–18,5)
Ácido úrico (mg/dL)	6,24 ± 1,49 (1,90–10,60)
ALT (U/L)	16,74 ± 21,86 (1,00–229,00)
Albumina (g/dL)	3,62 ± 0,75 (1,80–5,30)
Na (mmol/L)	134,66 ± 11,20 (14,00–146,00)
K (mmol/L)	5,72 ± 5,31 (3,00–7,70)
Ca (mg/dL)	8,40 ± 0,89 (5,10–10,50)
PCR (mg/L)	3,75 ± 5,96 (0,05–34,00)
TSH (µIU/mL)	15,05 ± 46,39 (0,12–491,00)
PTH (pg/mL)	686,00 ± 491,63 (32,30–2000,00)
Ferritina (ng/mL)	562,11 ± 568,08 (8,30–4511,00)
Fe (µg/dL)	48,42 ± 21,75 (9,00–142,00)
SII	1137,89 ± 2983,13 (0,00–33857,05)
NLR	5,02 ± 8,07 (0,25–79,11)
PLR	157,77 ± 115,57 (4,09–1126,32)
CAR	1,20 ± 2,06 (0,01–11,90)
HALP	0,41 ± 1,10 (0,00–12,77)
PNI	36,16 ± 7,60 (28,00–90,00)

Abreviaturas – DM: diabetes mellitus; HA: hipertensão arterial; WBC: leucócitos; ALT: alanina aminotransferase; Na: sódio; K: potássio; Ca: cálcio; PCR: proteína C-reativa; TSH: hormônio estimulador da tireoide; PTH: paratormônio; Fe: ferro; SII: índice de inflamação imune sistêmica; NLR: razão neutrófilos/linfócitos; PLR: razão plaquetas/linfócitos; CAR: razão PCR/albumina; HALP: hemoglobina × albumina × linfócitos / plaquetas; PNI: índice prognóstico nutricional.

**Tabela 2:** Desfechos de sobrevida de acordo com a idade e o sexo dos pacientes

Parâmetros		Sobreviventes		Mortalidade	
		N	%	N	%
Sexo	Feminino	53	74,6	18	25,4
	Masculino	40	62,5	20	37,5
				X^2^: 1,785	p = 0,181
Comorbidades	Presente	54	73,0	20	27,0
	Ausente	39	63,9	22	36,1
				X^2^: 0,888	p = 0,346

As análises univariadas dos parâmetros laboratoriais com potencial impacto na sobrevida dos pacientes estão apresentadas na Tabela 3. Idade, albumina, cálcio (Ca), proteína C-reativa (PCR), contagem de leucócitos (WBC), neutrófilos, hematócrito, hemoglobina, paratormônio (PTH), SII, NLR, CAR e PNI apresentaram associação estatisticamente significativa (p = 0,019; p = 0,008; p = 0,031; p <0,001; p <0,001; p = 0,001; p = 0,022; p = 0,001; p = 0,012; p = 0,016; p = 0,016; p <0,001; e p = 0,010, respectivamente).

**Tabela 3:** Distribuição da sobrevida de acordo com os parâmetros laboratoriais dos pacientes

Parâmetros	Status de sobrevida	N	Média ± DP	t	df	p
Idade (anos)	Sobreviventes	93	53,16 ± 17,34	–2,389	**98,4**	**0,019**
	Mortalidade	42	59,81 ± 13,76			
Kt/V	Sobreviventes	93	1,72 ± 0,36	0,580	133,0	0,563
	Mortalidade	42	1,69 ± 0,27			
Glicose (mg/dL)	Sobreviventes	93	135,53 ± 110,56	–0,917	133,0	0,361
	Mortalidade	42	153,00 ± 81,54			
Ureia (mg/dL)	Sobreviventes	93	113,28 ± 35,97	0,470	133,0	0,639
	Mortalidade	42	109,57 ± 54,23			
Creatinina (mg/dL)	Sobreviventes	93	7,41 ± 2,86	0,066	45,0	0,948
	Mortalidade	42	7,32 ± 8,79			
Ácido úrico (mg/dL)	Sobreviventes	93	6,23 ± 1,51	–0,044	133,0	0,965
	Mortalidade	42	6,25 ± 1,44			
ALT (U/L)	Sobreviventes	93	14,80 ± 12,13	–1,123	44,26	0,268
	Mortalidade	42	21,12 ± 35,12			
Albumina (g/dL)	Sobreviventes	93	3,76 ± 0,48	2,745	**48,5**	**0,008**
	Mortalidade	42	3,29 ± 1,08			
Na (mmol/L)	Sobreviventes	93	133,98 ± 13,15	–1,051	133,0	0,295
	Mortalidade	42	136,17 ± 4,30			
K (mmol/L)	Sobreviventes	93	5,35 ± 0,89	–0,813	41,3	0,421
	Mortalidade	42	6,54 ± 9,44			
Ca (mg/dL)	Sobreviventes	93	8,51 ± 0,90	2,185	**133,0**	**0,031**
	Mortalidade	42	8,15 ± 0,84			
Fe (µg/dL)	Sobreviventes	93	47,94 ± 22,46	–0,382	133,0	0,703
	Mortalidade	42	49,49 ± 20,29			
PCR (mg/L)	Sobreviventes	93	1,98 ± 3,74	–4,455	**49,6**	**<0,001**
	Mortalidade	42	7,66 ± 7,87			
WBC (×103/µL)	Sobreviventes	93	7,32 ± 2,57	–3,848	**45,9**	**<0,001**
	Mortalidade	42	11,65 ± 7,09			
Contagem de linfócitos (×10^3^/µL)	Sobreviventes	93	1,60 ± 0,67	–0,240	133,0	0,811
	Mortalidade	42	1,64 ± 1,18			
Contagem de neutrófilos (×10^3^/µL)	Sobreviventes	93	4,71 ± 2,05	–3,537	**44,6**	**0,001**
	Mortalidade	42	8,40 ± 6,62			
Hematócrito (%)	Sobreviventes	93	34,32 ± 5,30	2,320	**133,0**	**0,022**
	Mortalidade	42	32,03 ± 5,30			
Hemoglobina (g/dL)	Sobreviventes	93	10,94 ± 1,67	3,342	**133,0**	**0,001**
	Mortalidade	42	9,92 ± 1,57			
Contagem de plaquetas (×10^3^/µL)	Sobreviventes	93	214,00 ± 73,93	0,544	133,0	0,587
	Mortalidade	42	205,58 ± 100,82			
TSH (µIU/mL)	Sobreviventes	93	17,22 ± 79,81	0,542	51,0	0,590
	Mortalidade	42	0,73 ± 0,36			
PTH (pg/mL)	Sobreviventes	93	744,49 ± 546,75	2,545	**126,7**	**0,012**
	Mortalidade	42	556,48 ± 306,91			
Ferritina (ng/mL)	Sobreviventes	93	521,02 ± 479,11	–1,253	133,0	0,212
	Mortalidade	42	653,12 ± 726,19			
SII	Sobreviventes	93	725,32 ± 585,21	–2,435	**133,0**	**0,016**
	Mortalidade	42	2051,44 ± 5203,40			
NLR	Sobreviventes	93	3,38 ± 2,63	–2,518	**42,47**	**0,016**
	Mortalidade	42	8,60 ± 13,30			
PLR	Sobreviventes	93	150,29 ± 73,29	–0,846	47,60	0,400
	Mortalidade	42	174,17 ± 176,08			
CAR	Sobreviventes	93	0,58 ± 1,18	–4,511	**38,45**	**<0,001**
	Mortalidade	42	2,84 ± 2,87			
HALP	Sobreviventes	93	0,45 ± 1,30	0,655	130,0	0,514
	Mortalidade	42	0,32 ± 0,27			
PNI	Sobreviventes	93	37,65 ± 4,81	2,708	**44,05**	**0,010**

Abreviaturas – ALT: alanina aminotransferase; Na: sódio; K: potássio; Ca: cálcio; PCR: proteína C-reativa; WBC: contagem de leucócitos; TSH: hormônio estimulante da tireoide; PTH: paratormônio; Fe: ferro; SII: índice de inflamação imune sistêmica; NLR: razão neutrófilos/linfócitos; PLR: razão plaquetas/linfócitos; CAR: razão proteína C-reativa/albumina; HALP: hemoglobina × albumina × linfócitos / plaquetas; PNI: índice prognóstico nutricional.

Em consonância com o objetivo do estudo, a associação entre índices de fácil acesso que refletem o estado inflamatório e nutricional e a mortalidade em pacientes em HD foi avaliada por meio de análise de regressão logística multivariável. As variáveis incluídas no modelo multivariável foram selecionadas com base na relevância clínica e na significância estatística observada nas análises univariadas. O modelo foi ajustado para sexo e presença de comorbidades.

Antes da construção do modelo, a multicolinearidade entre as variáveis candidatas foi avaliada utilizando matrizes de correlação, VIF e estatísticas de tolerância. Para reduzir redundâncias e melhorar a estabilidade do modelo, índices compostos (como PNI e CAR) e seus componentes individuais (por exemplo, albumina, PCR, WBC) não foram inseridos simultaneamente no mesmo modelo, e as variáveis colineares foram excluídas de forma correspondente.

Dado o tamanho amostral limitado, os achados do modelo multivariável devem ser interpretados como associativos, e não preditivos, não tendo sido realizada avaliação formal do desempenho diagnóstico ou preditivo.

No modelo final, a CAR apresentou associação positiva com a mortalidade (*odds ratio* ajustada [aOR] = 1,374; intervalo de confiança [IC] de 95%: 1,011–1,869). Os níveis de PNI e hemoglobina apresentaram associação negativa com a mortalidade: PNI, aOR = 0,861 (IC 95%: 0,775–0,956); hemoglobina, aOR = 0,725 (IC 95%: 0,532–0,988). Não foram observadas associações significativas entre idade (aOR = 1,011; IC 95%: 0,980–1,043) ou WBC (aOR = 1,110; IC 95%: 0,943–1,307) e mortalidade. O ajuste global do modelo foi estatisticamente significativo (χ^2^(5) = 51,0; p <0,001), com R^2^ de Nagelkerke de 0,456 (Tabela 4). Utilizando o ponto de corte estabelecido, a classe positiva foi definida como “óbito”. O modelo classificou corretamente 91,4% dos sobreviventes (85/93) e 59,5% dos óbitos (25/42), com acurácia global de 82% (0,82). A sensibilidade foi de 0,60, a especificidade de 0,91 e a área sob a curva (AUC) foi de 0,87 (Tabela 5).

**Tabela 4:** Análise de regressão logística multivariada para mortalidade

Preditor	β	Valor de p	aOR	IC 95%	
				Inferior	Superior
Intercepto	5,834	0,023	341,625	2,221	52547,153
Idade (anos)	0,011	0,487	1,011	0,980	1,043
CAR	**0,318**	**0,043**	**1,374**	**1,011**	**1,869**
PNI	**–0,151**	**0,005**	**0,861**	**0,775**	**0,956**
Hemoglobina (g/dL)	**–0,322**	**0,042**	**0,725**	**0,532**	**0,988**
Leucócitos (×10^9^/L)	0,104	0,210	1,110	0,943	1,307
**Ajuste do modelo** Desvio (*Deviance*): 109 **Pseudo R** ^2^ Nagelkerke: 0,456 **Teste global do modelo** [X^2^(df), p]: 51,0 (5), <0,001					

Abreviaturas – CAR: razão proteína C-reativa/albumina; PNI: índice prognóstico nutricional. Notas – Variável dependente: mortalidade (não sobreviventes: 42; sobreviventes [referência]: 93). O modelo foi estimado com N = 135. aOR = *odds ratio* ajustada; as aORs foram ajustadas por sexo e presença de comorbidades (variáveis de controle).

**Tabela 5:** Desempenho preditivo do modelo desenvolvido (tabela de classificação)

Observado	Previsto		Acurácia (%)
	Sobreviventes	Não sobreviventes	
Sobreviventes	85	8	91,4
Não sobreviventes	17	25	59,5
Acurácia global	0,82		
Especificidade	0,91		
Sensibilidade	0,60		
AUC	0,87		

## Discussão

Neste estudo, a relação entre parâmetros clínicos e laboratoriais e a sobrevida foi avaliada em 135 pacientes submetidos a tratamento por HD. As análises univariadas revelaram associações significativas entre sobrevida e idade, albumina, cálcio, PCR, WBC, neutrófilos, hematócrito, hemoglobina, PTH, SII, NLR, CAR e níveis de PNI. Na análise de regressão logística multivariada, CAR, PNI e hemoglobina emergiram como preditores independentes de sobrevida. Enquanto níveis baixos de hemoglobina estiveram associados ao aumento da mortalidade, valores mais elevados de CAR e níveis mais baixos de PNI estiveram relacionados a um maior risco de mortalidade. O modelo estabelecido demonstrou desempenho preditivo clinicamente relevante, com alta especificidade (91%) e acurácia global de 82%.

O SII emergiu recentemente como um biomarcador relevante para a predição da mortalidade em pacientes em HD. Estudos relataram que níveis elevados de SII estão independentemente associados tanto à mortalidade por todas as causas quanto à mortalidade por doença cardiovascular e infecções^16,17^.

Além disso, diversos estudos compararam múltiplos índices inflamatório-nutricionais em populações em HD, relatando que o SII pode apresentar desempenho prognóstico relativamente mais forte para mortalidade quando avaliado juntamente com índices como NLR, PLR e CAR. No entanto, evidências provenientes de coortes retrospectivas menores que avaliam esses índices de forma concomitante sugerem que seu valor preditivo não é consistente entre diferentes populações e deve ser interpretado como exploratório e específico de cada coorte, e não como universalmente aplicável^18,19^.

Em nosso estudo, o SII esteve associado à sobrevida nas análises univariadas, mas não se mostrou um preditor independente nas análises multivariadas. Embora o valor prognóstico independente do SII tenha sido frequentemente enfatizado na literatura, essa discrepância pode ser atribuída a diferenças nos perfis dos pacientes, nas características da amostra ou nas variáveis incluídas no modelo.

A inflamação desempenha um papel significativo na desnutrição em pacientes em HD; portanto, a CAR atua como um marcador mais dinâmico, ao refletir tanto os processos inflamatórios quanto o *wasting* proteico-energético. Um estudo de Yang et al.^20^ demonstrou que a CAR supera outros biomarcadores, incluindo a albumina, na predição de inflamação sistêmica e complicações relacionadas. Além disso, a literatura destaca a CAR como um indicador prognóstico potencialmente relevante de mortalidade em pacientes em HD. Em uma coorte retrospectiva de pacientes incidentes em HD, níveis mais elevados de CAR foram relatados como independentemente associados ao aumento da mortalidade em um ano, sustentando o papel do desequilíbrio entre inflamação e nutrição nos desfechos de sobrevida^21^. Em nosso estudo, a CAR também emergiu como um preditor independente nas análises multivariadas e esteve entre os determinantes mais fortes da sobrevida. Esses achados são consistentes com relatos prévios e sugerem que a CAR representa um marcador prognóstico valioso para uso clínico na população em HD.

NLR e PLR são biomarcadores facilmente acessíveis da resposta inflamatória, e suas associações com mortalidade em pacientes em HD foram examinadas em diversos estudos. Essas investigações relataram que tanto valores elevados de NLR quanto de PLR estão significativamente associados à pior sobrevida^22^. Em nosso estudo, o NLR esteve significativamente associado à sobrevida nas análises univariadas, mas não manteve significância prognóstica independente após o ajuste multivariado, enquanto o PLR não esteve associado à sobrevida em análises univariadas ou multivariadas. Embora estudos anteriores tenham relatado associações entre níveis elevados de NLR e PLR e piores desfechos de sobrevida em populações em HD, nossos achados sugerem que a contribuição prognóstica desses índices pode ser dependente do contexto e sofrer atenuação quando variáveis clínicas e laboratoriais mais robustas são consideradas^23^.

Em um estudo prospectivo que avaliou aproximadamente 763 pacientes incidentes em HD, aqueles com escores HALP baixos apresentaram risco significativamente maior tanto de eventos cardiovasculares quanto de mortalidade por todas as causas em comparação com pacientes com escores elevados^24^. Além de outros índices inflamatório-nutricionais, o escore HALP foi explorado em populações em HD de manutenção, com estudos retrospectivos sugerindo uma associação potencial entre valores mais elevados e menor mortalidade por todas as causas. No entanto, essa associação não foi observada em nosso estudo, o que pode estar relacionado a diferenças nos perfis dos pacientes, na carga de comorbidades ou nas características clínicas, ressaltando a necessidade de validação específica por coorte do HALP como marcador prognóstico^25^. De forma semelhante, outro estudo recente relatou que níveis baixos de PNI estiveram associados a um maior risco de mortalidade em cinco anos em pacientes em HD, sugerindo que esse índice poderia atuar como um preditor independente de sobrevida em longo prazo^26^. Em nosso estudo, os níveis de PNI estiveram significativamente associados à sobrevida, com valores mais elevados de PNI relacionados ao menor risco de mortalidade. Esses achados são consistentes com relatos anteriores e sustentam a noção de que o PNI representa um biomarcador importante a ser considerado na avaliação prognóstica de pacientes em HD.

Nessa população, categorias de hemoglobina <9,0; 9,0–9,9; e ≥13,0 g/dL foram associadas a um risco significativamente aumentado de mortalidade em comparação com a faixa de referência de 10,0–10,9 g/dL. Além disso, a categoria de 12,0–12,9 g/dL também pode conferir risco elevado, dependendo da história clínica do paciente (por exemplo, determinadas comorbidades). Esses achados sustentam a noção de que uma faixa aproximada de hemoglobina de 10–12 g/dL é ideal em pacientes em HD^27,28^. Em nossa coorte, os níveis de hemoglobina foram significativamente mais elevados entre os sobreviventes (10,94 ± 1,67 g/dL) e mais baixos entre aqueles que evoluíram para óbito (9,92 ± 1,57 g/dL; t = 3,342; gl = 133; p = 0,001). A diferença média de aproximadamente 1,0 g/dL indica que o grupo que foi a óbito estava mais próximo do limiar <10 g/dL, enquanto os sobreviventes situavam-se na faixa de 10,0–10,9 g/dL. Quando nossos dados são considerados em conjunto com os achados publicados, as evidências que associam níveis baixos de hemoglobina (<10 g/dL) ao aumento da mortalidade são reforçadas. Esses resultados sugerem que, na prática clínica, é racional direcionar os níveis de hemoglobina para uma faixa aproximada de 10–12 g/dL, evitando a supercorreção (≥13 g/dL) e considerando as comorbidades. Embora nossa amostra não tenha incluído pacientes suficientes para avaliar riscos nos níveis mais elevados, os dados disponíveis sustentam claramente o risco associado a níveis mais baixos de hemoglobina.

As limitações do nosso estudo incluem seu desenho retrospectivo, o cenário de centro único e o tamanho amostral relativamente pequeno, que restringem a generalização dos achados e introduzem potencial viés de seleção. Além disso, alguns parâmetros (por exemplo, PTH, ALP) foram excluídos da análise devido à ausência de mensuração rotineira ou à variabilidade relacionada ao tratamento. Ademais, o desenho transversal impediu a avaliação de mudanças temporais nos biomarcadores e não permitiu a determinação de relações causais. Por fim, apenas valores basais foram considerados, e o impacto de alterações dinâmicas dos biomarcadores durante o seguimento de longo prazo sobre o prognóstico não pôde ser avaliado.

## Conclusão

Em conclusão, o presente estudo demonstrou que os níveis de CAR, PNI e hemoglobina estiveram associados à sobrevida na análise de regressão logística multivariável. Esses achados sugerem que biomarcadores que refletem o estado inflamatório, o estado nutricional e os parâmetros hematológicos podem estar relacionados ao prognóstico em pacientes em HD. Destaca-se a utilidade potencial de parâmetros de uso rotineiro e de fácil obtenção, como CAR e PNI, na avaliação prognóstica. No entanto, considerando o desenho do estudo e seu tamanho amostral, são necessários estudos adicionais, de maior escala e prospectivos, para esclarecer o papel desses biomarcadores na tomada de decisão clínica.

## Disponibilidade de Dados

Os dados não estão publicamente disponíveis devido a restrições institucionais, mas podem ser fornecidos mediante solicitação razoável ao autor correspondente.
